# Concentration-Dependent Effects of Boric Acid on Osteogenic Differentiation of Vascular Smooth Muscle Cells

**DOI:** 10.1007/s12011-024-04204-6

**Published:** 2024-05-03

**Authors:** Osama Al Khalif, Gülay Sezer

**Affiliations:** 1 Department of Pharmacology, Faculty of Medicine, Erciyes University, 38039 Kayseri, Turkey; 2 Genkök Genome and Stem Cell Center, Erciyes University, 38039 Kayseri, Turkey

**Keywords:** Boron, Boric acid, Vascular calcification, Vascular smooth muscle cells, Nrf2

## Abstract

Vascular calcification can be triggered by oxidative stress and inflammation. Although boron possesses antioxidant and anti-inflammatory properties, its effect on osteogenic differentiation of vascular smooth muscle cells (VSMCs) has yet to be examined. Therefore, we aimed to investigate the effect of boric acid (BA), the main form of boron in body fluids, on the osteogenic differentiation of VSMCs. Following the isolation of VSMCs, the effects of BA on cell proliferation were determined by MTT. The impact of various BA concentrations on the osteogenic differentiation of VSMCs was evaluated by Alizarin red S and alkaline phosphatase (ALP) stainings and the o-cresolphthalein complexone method. In addition, mRNA expressions of osteogenic-related (Runx2 and ALP) and antioxidant system-related genes (Nrf2 and Nqo1) were detected using qRT-PCR analysis. BA treatments did not alter the proliferation of VSMCs. Osteogenic differentiation of VSMCs treated with 100 and 500 μM BA (moderate and high plasma concentrations) was no different from untreated cells. However, increased osteogenic differentiation was observed with the lowest blood level (2 μM) and extremely high BA concentration (1000 μM). Consistent with these results, mRNA expression of Runx2 increased with 2 and 1000 μM BA treatments, while Nrf2 and Nqo1 expressions increased significantly with 100 and 500 μM BA. BA has different effects on VSMCs at various concentrations. The low blood level and too high BA concentration appear detrimental as they increase the osteogenic differentiation of VSMCs in vitro. We propose to investigate BA’s effects and mechanism of action on vascular calcification in vivo.

## Introduction

Cardiovascular diseases are the leading cause of morbidity and mortality worldwide [1]. Vascular calcification is one of the most important determinants of cardiovascular risk, characterized by thickening and loss of elasticity of muscular artery walls. Vascular calcification is highly associated with cardiovascular disease mortality, especially in high-risk patients with diabetes and chronic kidney disease. Calcification of both intimal and medial layers is an active and tightly regulated process driven primarily by vascular smooth muscle cells. (VSMCs) [2, 3].

VSMCs play a critical role in vascular calcification as they can change their phenotype into osteoblast-like cells and secrete extracellular matrix. This transformation process is accompanied by the accumulation of calcium salts (such as hydroxyapatite) in the extracellular matrix, mimicking bone formation [4]. The osteogenic differentiation of VSMCs can be stimulated by factors like oxidative stress, inflammatory mediators, and elevated levels of inorganic phosphate (mineral imbalance). Oxidative stress plays an important role in the pathogenesis of vascular calcification and contributes to atherogenesis. Excessive reactive oxygen species (ROS) contribute to the activation of signal transduction pathways in calcification of VSMCs [3, 5]. Differentiation of VSMCs to an osteoblast-like phenotype is associated with the expression of osteogenesis markers such as runt-related transcription factor 2 (Runx2), collagen type I (Col1), osteocalcin, and alkaline phosphatase (ALP) [2, 5]. Runx2, as an osteogenic transcription factor, is pivotal in regulating the expression of osteoblast marker genes involving ALP, Col1, and osteocalcin [5].

Boron is an essential trace element for maintaining the health of all living things. Boron-based compounds also have widespread industrial and commercial use. Boron is found in various foods (e.g., fruits and legumes) and beverages but is also widely used as a dietary supplement (e.g., boron ascorbate and calcium fructoborate). Although boron is mainly taken orally through various foods, it also enters the body through the dermal and inhalation routes [6, 7]. Boron supports bone health, improves brain function, regulates immune and inflammatory responses, and influences how the body responds to oxidative stress. Boron can play a crucial role in metabolism by regulating enzymes and hormones [6–8]. Numerous studies have substantiated the beneficial effect of boron on the cardiovascular system [7, 8]. A significant decrease in serum boron concentrations was noticed in atherosclerotic patients compared to healthy subjects [9]. Moreover, patients with stable angina who received boron supplementation experienced a decrease in C-reactive protein (CRP), low-density lipoprotein (LDL), and total cholesterol levels, along with an improvement in high-density lipoprotein (HDL) levels [10]. It has been reported that regular boron supplementation to healthy individuals provides a significant decrease in LDL, total cholesterol, triglyceride, CRP, interleukin-1β (IL-1β), interleukin-6 (IL-6), and monocyte chemoattractant protein-1 (MCP-1) levels and an increase in HDL [11]. Another study reported that higher plasma boron levels were linked to a healthy diet, associated with reduced body mass index and a more favorable cardiometabolic risk profile [12]. After consumption of boron compounds, boron undergoes hydrolysis, primarily transforming into boric acid (BA), the primary form of boron in the bloodstream and other body fluids [8]. Blood boron levels have been reported to range from 21 to 1232 ng boron/g blood (equivalent to 2 to 120.8 µM BA) depending on the individual’s dietary habits and geographical location [12, 13]. It has been reported that daily boron intakes for adults eating standard diets can be estimated to be roughly 1–3 mg/day [7, 13]. However, in countries with high boron reserves, especially China and Turkey, environmental and occupational boron exposure may be approximately 20 to 40 times higher [14]. Therefore, in the present study, we investigated the effects of 2, 100, 500, and 1000 µM BA on osteogenic differentiation of VSMCs. It is reported that boron plays a vital role in osteogenesis and bone maintenance, and its deficiency negatively affects bone development and regeneration [7]. Boron has also been reported to enhance osteogenic differentiation of bone marrow stromal cells (BMSCs) [15].

Nuclear factor erythroid 2-related factor 2 (Nrf2) is an ubiquitously expressed key transcription factor, plays a crucial role in combating oxidative stress, and regulates various antioxidant enzymes such as NAD(P)H quinone oxidoreductase-1 (Nqo1), heme oxygenase-1 (HO-1), glutathione reductase, and peroxiredoxins [16–18]. Boron compounds and BA have antioxidant effects in various tissues through their effects on antioxidant systems such as Nrf2 and enzymes such as malondialdehyde (MDA), superoxide dismutase (SOD), catalase (CAT), glutathione (GSH), and glutathione peroxidase (GPx) [7, 19, 20]. A relationship between decreased boron concentration and atherosclerosis has been demonstrated [9], but to date, the effects of boron on osteogenic differentiation of VSMCs have not been reported. In this study, we aimed to evaluate the effect of BA on vascular calcification and Nrf2 expression under in vitro conditions.

## Materials and Methods

### Preparation of Boric Acid Solution

Molecular biology grade BA (Sigma-Aldrich, USA) was dissolved in ultrapure water (Gibco, USA) mixed until completely dissolved. The solution was sterilized by filtration through a 0.22-μm syringe filter to obtain a 100 mM sterile stock solution. The stock solution prepared BA at final concentrations in the growth medium.

### Isolation and Culture of VSMCs

VSMCs were isolated from the thoracic aorta of 4- to 6-week-old Sprague–Dawley rats. This study was performed in line with the principles of the Declaration of Helsinki. Approval was granted by the Ethics Committee of Erciyes University (date 03.02.2021/No 21/35). VSMCs were isolated according to the method in the literature and maintained in Dulbecco’s modified Eagle’s high glucose (DMEM, Sigma, St. Louis, MO, USA) growth medium supplemented with 10% fetal bovine serum (FBS, Gibco, USA), 1% glutamax (Gibco, USA), and 1% penicillin/streptomycin (Gibco, Paisley, UK) in a humidified atmosphere of air/5% CO_2_ at 37 °C [21]. The medium was changed every 2–3 days. VSMCs were used between passages 3 and 6.

### Characterization of VSMCs

VSMCs were identified by their typical hill-and-valley growth patterns and immunofluorescence staining of α-smooth muscle actin (α-SMA) protein. Briefly, VSMCs seeded on glass coverslips were rinsed with phosphate-buffered saline (PBS) and then fixed with formaldehyde (10%) for 10 min. Next, VSMCs were permeabilized by 0.3% Triton-X solution for 3 min and then treated with 10% bovine serum albumin (BSA) for 2 h. For staining, α-SMA polyclonal antibody (E-AB-34268, Elabscience, USA, 1:240 µL) was diluted in 1% BSA, added to each coverslip, and incubated overnight at + 4 °C in the dark. On the subsequent day, the coverslips underwent a PBS wash and were incubated with an Alexa Fluor 488-goat anti-rabbit IgG (ab150077, Abcam, UK, 1:400 µL) secondary antibody diluted in 1% BSA. After 1 h of incubation, coverslips were rinsed with PBS and covered on a slide with a DAPI-containing medium, and images were obtained using immunofluorescence microscopy (Nikon Ni-E, Japan).

### Cell Proliferation

Cell proliferation was determined by MTT assay. VSMCs were seeded at 3000 cells/well in 96-well plates and left to attach overnight. Subsequently, cells were treated with different concentrations of BA (0, 1, 10, 20, 50, 100, 200, 500, and 1000 µM) and incubated at 37 °C for 24 and 72 h. Afterward, 10 μL MTT (3-(4,5-dimethyl-2-thiazolyl)-2,5-diphenyl-2-H-tetrazolium bromide) solution (5 mg/mL, Sigma-Aldrich, USA) was added to each well and incubated for an additional 3 h. The medium was discarded, and 100 μL of dimethyl sulfoxide was added to each well. Then, the absorbance was measured at 560 nm on a spectrophotometer (Glomax Multimode Plate Reader, Promega, USA). Results were standardized to the control group and presented as percent proliferation [22].

### Induction of Calcification

Cells were treated with different concentrations of BA to determine the effect of BA on osteogenic differentiation of VSMCs. VSMCs were seeded at 20,000 cells/well of a 12-well plate and randomly assigned into six groups. The groups consisted of a negative control group receiving growth medium only, a positive control group receiving osteogenic medium, and four treatment groups, each receiving a different concentration of BA (2, 100, 500, and 1000 μM) in the presence of osteogenic medium. The osteogenic medium was prepared by the addition of 10 mM β-sodium glycerophosphate (Sigma-Aldrich, USA) and 50 μg/mL ascorbic acid (Sigma-Aldrich, USA) to the growth medium [23]. The effect of BA on the calcification process was observed at two different time points (14 and 21 days). The day when the osteogenic medium was first applied was considered day 1. BA treatment and osteogenic media application were used simultaneously on the same day, and culture media were changed every 2–3 days. The effect of weak acid BA on the pH of the medium was examined by pH measurements 24 and 48 h after BA treatments.

### Determination of Calcification

The calcified matrix was evaluated by Alizarin red S staining. On days 14 and 21, VSMCs cultured in a 12-well plate were rinsed with PBS and subsequently fixed with 10% formaldehyde for 10 min at room temperature. Subsequently, the cells were rinsed with distilled water and stained with 2% Alizarin red solution (pH 4.2) for 30 min in the dark. Then, the plates were rewashed with distilled water, and images were taken under an inverted microscope (Leica DMi1, Germany). ImageJ program (NIH, Bethesda, MD) was used to quantify Alizarin red S-stained red areas in microscopic photo images. The staining area was measured in three experiments, using the percentage of areas in at least three randomly taken images from each well.

To measure the calcium concentrations in the cell layers, VSMCs were decalcified using 0.6 M hydrochloric acid for 1 day at + 4 °C. The supernatant was collected in centrifuge tubes and determined by the *o*-cresolphthalein complexone (Sigma-Aldrich, USA). When combined with calcium, this reagent produces a purple color, and its absorbance was measured at 560 nm by a plate reader. The cells were rinsed with cold PBS (without Ca2 + and Mg2 +) and scraped from the culture plate to measure the total protein content. According to the manufacturer’s instructions, the total protein content was calculated using the BCA protein assay kit (Pierce, USA). Calcium content was normalized to protein concentration and expressed as µg/mg protein.

### Alkaline Phosphatase (ALP) Staining

The enzymatic activity of ALP was assessed on days 14 and 21. The reaction of ALP enzyme with a mixture of nitro-blue tetrazolium chloride (NBT) and 5-bromo-4-chloro-3’-indolephosphate b-toluidine salt (BCIP) leads to the development of insoluble purple-black precipitates. VSMCs were fixed in 10% formaldehyde for approximately 10 min, then 500 µL of NBT/BCIP substrate solution (1-Step NBT/BCIP, Thermo Fisher, USA) was added to each well of 12-well plates and incubated in the dark for approximately 15 min. To remove excess dye, wells were rinsed, and ALP-stained blue-purple field images were randomly taken by an inverted microscope (Leica DMi1, Germany) and quantified using the ImageJ program. The staining area was measured in three experiments, using the percentage of areas in at least three randomly taken images from each well.

### RNA Extraction and Quantitative Real-Time PCR (qRT-PCR)

To extract total RNA from VSMCs, TRIzol reagent (TranszolUp, Transgenbiotech, China) was used following the manufacturer’s instructions. Subsequently, the extracted RNA was reverse transcribed using the OneScript Plus cDNA synthesis kit (ABM, Canada) according to the provided instructions.

Expression levels of osteogenic-related (Runt-related transcription factor 2; Runx2 and ALP) and antioxidant system-related genes (Nrf2 and Nqo1) were determined using qRT-PCR. For this analysis, predesigned FAM™-labeled TaqMan assays and TaqMan™ fast advanced master mix (Thermo Fisher, 4444557) were employed. The Step One Plus™ real-time PCR system (Thermo Fisher, USA) was used to run the PCR reactions. The mRNA levels of Runx2 (Rn01512298_m1), ALP (Rn01516028_m1), Nrf2 (Rn00582415-m1), and Nqo1 (Rn00566528_m1) were quantified using the 2^−ΔΔCT^ method, with beta-actin (Rn00667869_m1) serving as the reference gene for normalization.

### Statistical Analysis

The results are the mean ± standard deviation (SD) of three independent experiments for each assay. Statistical analyses were conducted using SPSS 22.0 (IBM, USA). The normality of the data was assessed using the Shapiro–Wilk normality test. Student’s *t*-test analyzed statistical differences between the two groups. For comparisons between multiple groups, one-way ANOVA was performed, followed by post hoc Tukey HSD based on homogeneity of variance analysis. Statistical significance was considered at *p* < 0.05.

## Results

### Characterization of VSMCs

**Fig. 1:**
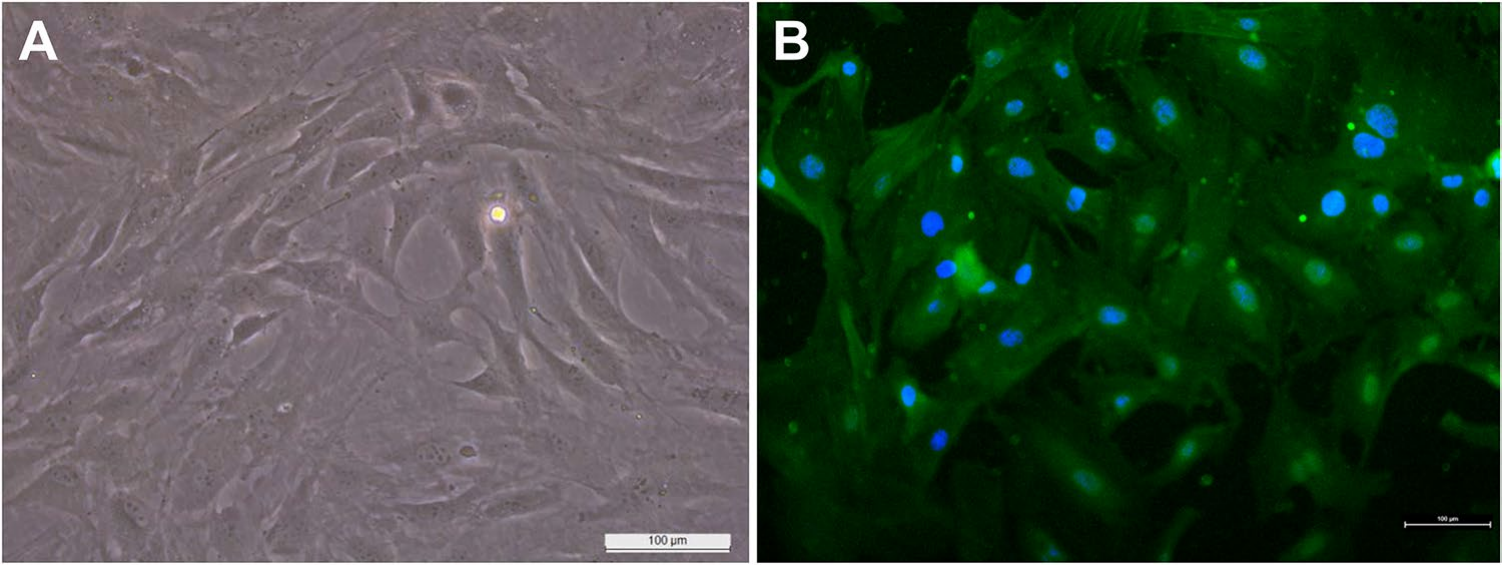
Characterization of the VSMCs by morphology and immunofluorescence staining. **a** Inverted microscope image of isolated VSMCs at passage 2 (× 20, scale bar: 100 μm); **b** immunofluorescence staining of isolated VSMCs for α-SMA (green) and DAPI (blue) (× 20, scale bar: 100 μm)

The morphology of cells was observed under an inverted microscope. As shown in Fig. 1a, the cells exhibited a spindle-shaped appearance with a characteristic “hill-and-valley” pattern at the confluence; this indicates that the isolated cells are VSMCs. To characterize VSMCs and determine their purity, immunofluorescence staining was performed for α-SMA (Fig. 1b). Of the 257 cells counted in 10 randomly picked images, 251 cells were determined to be stained with α-SMA. Based on this result, cells in culture were mainly composed of VSMCs, with a purity of 97.6%

### The Effect of BA on VSMC Proliferation

**Fig. 2:**
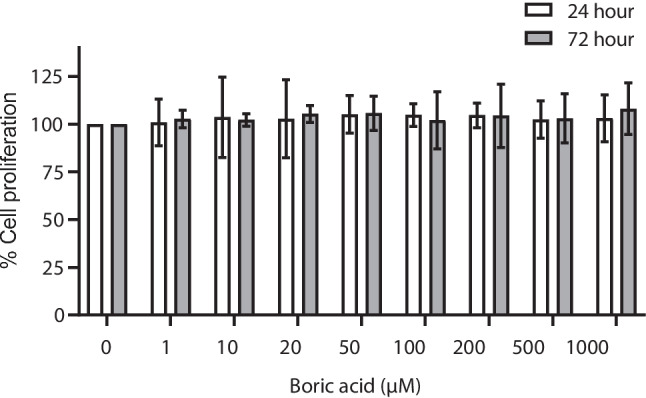
The effect of BA on the proliferation of VSMCs was measured by MTT assay at 24 and 72 h. Values are presented as mean ± SD, *n* = 3. *p < 0.05 compared to control

To investigate the effect of BA on VSMC proliferation, cells were treated with eight different concentrations (1–1000 μM) of BA for 24 and 72 h. BA had no significant effect on the proliferation of VSMCs compared with the control group (*p* > 0.05) (Fig. 2). In addition, it was observed that the pH values of BA-treated cells at 24- and 48-h incubation were not much different compared to the control group (Table 1).

**Table 1:** pH values of growth media in the presence of BA at 24 and 48 h

H	Negative control	Positive control	BA μM			
			2	100	500	1000
24 h	8.56	8.58	8.48	8.47	8.47	8.49
48 h	8.07	8.06	8.05	8.06	8.05	8.07

### Determination of Calcification

**Fig. 3:**
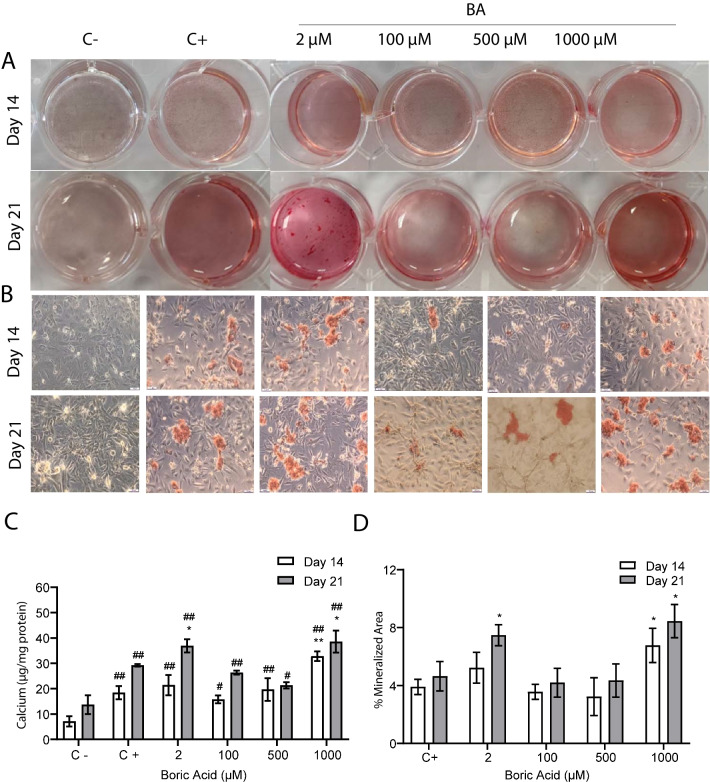
Alizarin red S staining images and calcium content of VSMCs cultured in culture medium (negative control group, C −), osteogenic medium (positive control group, C +), and osteogenic medium with BA for 14 and 21 days. **A** Photos of Alizarin red S-stained wells, **B** microscopic images of Alizarin red S-stained cells (scale bar: 100 μm, × 10), **C** quantification of mineralized areas stained with Alizarin red S (%), and **D** quantitative analysis of calcium content. Bars represent the mean ± SD, *n* = 3. ^*^p < 0.05 and ^**^p ≤ 0.005 compared to C + , ^#^*p* < 0.05 and ^##^*p* ≤ 0.005 compared C − group

On 14 and 21 days of treatment, no Alizarin red staining was observed in the negative control group, while calcium deposits were observed in the positive control and BA-treated groups (Fig. 3 A, B). Quantitative analysis of calcium revealed a significant increase in the positive control group compared to the negative control group on days 14 and 21 (*p*: 0.005 and *p*: 0.000, respectively). On day 14, a significant increase in calcium deposit was observed only in cells treated with 1000 µM BA compared to the positive control group (*p*: 0.032). On day 21, a significant increase in calcium deposits was observed in both the 2 and 1000 µM BA-treated groups compared to the positive control group (*p*: 0.007 and *p*: 0.042, respectively, Fig. 3C). Per the results of Alizarin red staining, a significant increase in calcium amount was determined in the 1000 µM BA-treated group compared to the positive control group on day 14 (*p*: 0.001). On day 21, significant increases in calcium levels were observed in the 2- and 1000-µM BA-treated groups compared to the positive control group (*p*: 0.039 and *p*: 0.011, respectively). In contrast, there was no significant difference between the 100-µM and 500-µM BA-treated groups compared to the positive control group at both periods (Fig. 3D).

### ALP Activity

**Fig. 4:**
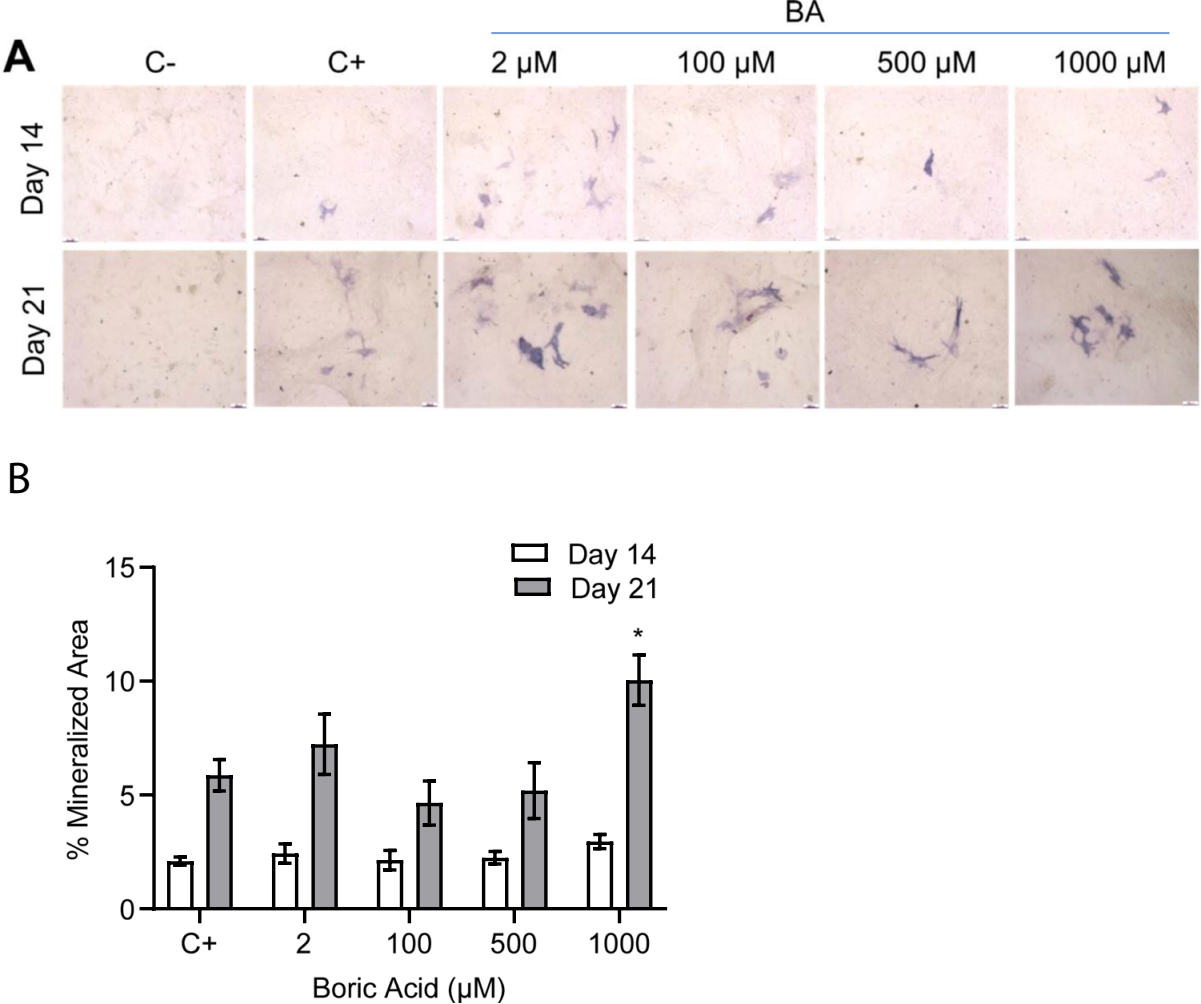
ALP staining images and stained area of VSMCs cultured in culture medium (negative control group, C −), osteogenic medium (positive control group, C +), and osteogenic medium + BA (2, 100, 500, and 1000 μM) for 14 and 21 days. **A** Microscopic images of ALP-stained cells (× 10, scale bar: 100 μm, and **B** quantification by ImageJ-based analysis for % mineralized area on days 14 and 21. Values represent the mean ± SD, *n* = 3. ^*^p < 0.05 compared to positive control

ALP activity was examined by immunohistochemical staining with NBT/BCIP dye on days 14 and 21. The results showed no notable change in ALP activity in the BA-treated groups compared to the positive control group on day 14. However, on day 21, there was a significant increase in the group treated with 1000-µM BA compared to the positive control group (*p*: 0.006) (Fig. 4b).

### Gene Expression Assay

**Fig. 5:**
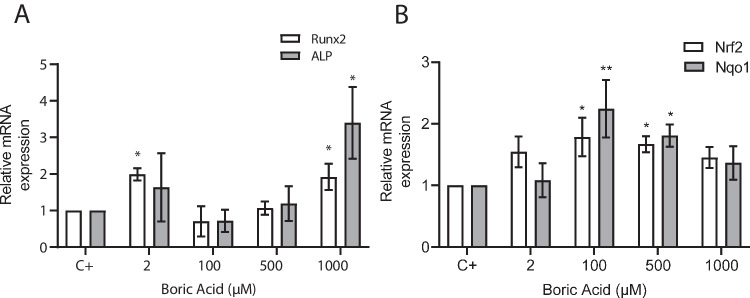
Relative mRNA expressions of **A** Runx2 and ALP, and **B** Nrf2 and Nqo1 of VSMCs cultured with osteogenic medium (C +) and osteogenic medium + BA (2, 100, 500, and 1000 μM) for 21 days. Values are presented as mean ± SD, *n* = 3. *p < 0.05, p** < 0.005 compared to C + group

Osteogenic differentiation of VSMCs is associated with increased mRNA expression of osteoblast markers (Runx2 and ALP). Similar to results obtained in Alizarin red S staining and calcium measurements, there was a significant increase in mRNA expression of Runx2 in the groups treated with BA 2 and 1000 μM compared to the positive control group cells (*p*: 0.008 and *p*: 0.013, respectively). No significant difference was observed in the 100- and 500-μM BA-treated cells compared to the control cells (*p*: 0.679 and *p*: 0.997, respectively) (Fig. 5A).

Similarly, mRNA expression of ALP increased significantly in the 1000-μM BA-treated group compared to the control group (*p*: 0.008). There was no difference in ALP expression in the 2-, 100-, and 500-μM BA-treated groups compared to the control group (*p* > 0.05) (Fig. 5A).

Nrf2 and Nqo1mRNA expressions increased significantly in the groups treated with 100- and 500-μM BA compared to the control group (*p* < 0.05). However, there was no significant change in the mRNA expressions of Nrf2 and Nqo1 in groups treated with 2- and 1000-μM BA compared to the positive control group (*p* > 0.05) (Fig. 5B).

## Discussion

This study demonstrated that moderate and high plasma concentrations of BA (100 and 500 μM, respectively) did not affect VSMC calcification. In comparison, low plasma concentration (2 μM) and extremely high concentration (1000 μM) caused a significant increase in the calcification process. The concentration-dependent effect of BA may be substantial as plasma boron concentrations may vary depending on the individual’s dietary habits, boron concentrations in the soil, and use of boron dietary supplements [12, 13]. In addition, environmental and occupational boron exposure is extremely high around the boron mining areas. The highest average total daily boron exposure levels in mining areas or processing facilities in China and Turkey were reported to be 41.2 and 47.17 mg/day, respectively [14]. Furthermore, since boron is not accepted as an essential element, human requirements for boron have not yet been defined. However, while the tolerable upper limit for adults in the USA is reported to be approximately 20 mg/day [24], the European Food Safety Authority has set the upper intake level for adults at 10 mg/day [25]. Therefore, this study is crucial as it provides information on whether BA has any effect on the calcification of VCMCs and also reveals the concentration-dependent effects of BA on the calcification process.

To date, boron compounds have been shown to have many beneficial effects. Their anti-inflammatory [11, 19] and antioxidant effects [19, 20, 26, 27] make them important protective agents against many diseases, including the cardiovascular system [8]. Lower boron levels have been reported in the bloodstream of patients diagnosed with atherosclerosis compared to healthy individuals [9]. Additionally, 1 month of boron supplementations in healthy subjects resulted in a significant decrease in IL-1β, IL-6, CRP, and MCP-1 compared to the placebo group [11]. Similarly, boron supplementation caused a reduction in total cholesterol and LDL levels along with an increase in HDL levels [10, 11]. On the other hand, there is an association between high serum calcium and the development of vascular calcification in chronic kidney disease [2], and boron supplementation led to a decrease in urinary calcium excretion and an increase in plasma calcium levels [28]. Additionally, excess vitamin D has been shown to induce vascular calcification in animal models and VSMC culture [29, 30]. Boron has been shown to increase serum vitamin D levels, possibly by decreasing catabolism [31]. Therefore, excessive boron consumption may increase calcium and active vitamin D levels, which may cause harmful effects such as vascular calcification. In addition, it was reported that plasma estradiol concentrations increased significantly due to boron supplementations of patients [32] and postmenopausal women [28]. In a clinical study, higher plasma estradiol levels were associated with reduced atherosclerosis progression in early postmenopausal women but increased progression in late postmenopausal women [33]. Another explanation for BA-induced vascular calcification may be due to the histone deacetylase inhibitor activity of boron compounds and BA [7, 34]. Several histone deacetylase inhibitors have been shown to promote osteoblast maturation by enhancing Runx2-dependent transcriptional activation [7]. Recently, butyrate, a histone deacetylase inhibitor, has been shown to accelerate osteogenic differentiation of VSMCs [35].

A relationship has been reported between the serum level of ALP, a cell membrane-associated enzyme that promotes vascular calcification, and the coronary artery calcification score in maintenance hemodialysis patients [36] and chronic kidney disease rats [34]. In the present study, BA at 100 and 500 μM did not cause a significant change in calcium accumulation in VSMCs but led to a substantial increase at extremely low (2 μM) and extremely high concentrations (1000 μM). In accordance, BA at 100 and 500 μM did not cause any significant increase in ALP and Runx2 mRNA expressions. Similar to our results, Hakki et al. demonstrated that the effects of BA on Runx2 mRNA and bone morphogenetic proteins (BMP)-4, -6, and -7 protein expressions during osteoblastic differentiation of MC3T3-E1 cells were time and dose dependent [37]. Ying et al. observed that calcium deposits increased in BMSCs treated with 1 and 10 ng/ml BA and significantly reduced in the 100 and 1000 ng/ml BA groups [15].

Oxidative stress is one of the key factors in stimulating the osteogenic differentiation of VSMCs, and it is closely related to atherosclerosis and chronic kidney disease [5]. Wei et al. demonstrated that high inorganic phosphate concentrations promote the accumulation of ROS in VSMCs and exacerbate calcification following Nrf2 knockdown in these cells [17]. As a critical mediator against ROS production, Nrf2 has been reported to ameliorate intracellular oxidative stress and calcium accumulation in VSMCs under high inorganic phosphate conditions [16]. Activation of the Nrf2 antioxidant signaling pathway has also been reported to protect against the onset and progression of vascular calcification associated with chronic kidney disease in rats by reducing intracellular oxidative stress [16]. Studies have shown that activation of the Nrf2 system by agents such as dimethyl fumarate or butyl hydroquinone reduced the calcification process in VSMCs [16, 17]. Indeed, the primary function of Nrf2 is to maintain cellular homeostasis by activating genes that encode cytoprotective and antioxidant enzymes, such as Nqo1 and HO-1 [18]. Activation of Nrf2 also leads to increased levels and/or activity of the key enzyme gamma-glutamylcysteine ligase, the rate-limiting enzyme for GSH synthesis [38]. It has been reported that boron activates the antioxidant system through the Nrf2 pathway to protect many tissues from oxidative damage. In a nitrite-induced hepatorenal dysfunction animal model, lithium borate significantly increased the Nrf2/HO-1 signaling pathway [26]. Another study reported that BA had an antioxidant effect due to increased Nrf2 and Nqo1 mRNA expressions in mouse embryonic fibroblast and human prostate cancer cells [39]. BA administration to ostrich chicks significantly increased Nrf2 and HO-1 mRNA expressions in kidney tissues and inhibited apoptosis [27]. Similar to our results, the expressions of Nrf2 and its direct target Nqo1 were reported to significantly increase in rats with chronic kidney disease to respond favorably to kidney injury and play a critical role in developing vascular calcification [16]. Our study observed that moderate plasma concentrations of BA enhanced the expression of antioxidant genes (Nrf2/Nqo1). The upregulation of Nrf2 and Nqo1 mRNA expressions explains why, despite calcification induction, Runx2 /ALP expressions were not significantly increased in BA 100- and 500-μM-treated groups compared to the control group. In addition, while there was no significant increase in mRNA expression of antioxidant genes in the groups administered 2 and 1000 μM BA, an increase in calcification was observed.

There are certain limitations to the present study. First, we did not examine whether BA causes calcification without beta-glycerophosphate in VSMCs. Therefore, we do not know whether BA induces calcification if there is no environmental calcification factor. Additionally, the present research was conducted using an in vitro model, which allows us to understand the effect of boron on the calcification process under specific conditions. However, in vivo vascular calcification is affected by numerous factors due to complex systemic feedback regulatory mechanisms. Therefore, performing this research in vivo allows us to investigate the effects of BA with all the factors that contribute to the calcification of blood vessels.

In conclusion, this is the first time the relationship between BA and the calcification of VSMCs has been investigated. Our study revealed the concentration-dependent effects of BA on the calcification of VSMCs. Low or extremely high plasma levels of BA led to a significant increase in the differentiation of VSMCs in vitro. However, it is interesting that medium and high plasma concentrations of BA did not alter the calcification profile of VSMCs in the osteogenic environment, consistent with their effects on antioxidant system-related gene expressions. Based on these results, we recommend additional studies on the impact and mechanism of action of different doses of boron on the calcification of blood vessels in vivo.

## Data Availability

The data supporting this study’s findings are available on reasonable request from the corresponding author [G.S.].

## References

[1] Vaduganathan M, Mensah GA, Turco JV, Fuster V, Roth GA (2022) The global burden of cardiovascular diseases and risk: a compass for future health. J Am Coll Cardiol 80(25):2361–2371. 10.1016/j.jacc.2022.11.005 36368511 10.1016/j.jacc.2022.11.005

[2] Chen NX, Moe SM (2012) Vascular calcification: pathophysiology and risk factors. Curr Hypertens Rep 14(3):228–237. 10.1007/s11906-012-0265-8 22476974 10.1007/s11906-012-0265-8 PMC3959826

[3] Durham AL, Speer MY, Scatena M, Giachelli CM, Shanahan CM (2018) Role of smooth muscle cells in vascular calcification: implications in atherosclerosis and arterial stiffness. Cardiovasc Res 114(4):590–600. 10.1093/cvr/cvy010 29514202 10.1093/cvr/cvy010 PMC5852633

[4] Petsophonsakul P, Furmanik M, Forsythe R, Dweck M, Schurink GW, Natour E, Reutelingsperger C, Jacobs M, Mees B, Schurgers L (2019) Role of vascular smooth muscle cell phenotypic switching and calcification in aortic aneurysm formation. Arterioscler Thromb Vasc Biol 39(7):1351–1368. 10.1161/atvbaha.119.312787 31144989 10.1161/ATVBAHA.119.312787

[5] Lee SJ, Lee IK, Jeon JH (2020) Vascular calcification-new insights into its mechanism. Int J Mol Sci 21(8):2685. 10.3390/ijms21082685 32294899 10.3390/ijms21082685 PMC7216228

[6] Khaliq H, Juming Z, Ke-Mei P (2018) The physiological role of boron on health. Biol Trace Elem Res 186:31–51. 10.1007/s12011-018-1284-3 29546541 10.1007/s12011-018-1284-3

[7] Pizzorno L (2015) Nothing boring about boron. Integr Med (Encinitas) 14:35–48 26770156 PMC4712861

[8] Donoiu I, Militaru C, Obleagă O, Hunter JM, Neamţu J, Biţă A, Scorei IR, Rogoveanu OC (2018) Effects of boron-containing compounds on cardiovascular disease risk factors - a review. J Trace Elem Med Biol 50:47–56. 10.1016/j.jtemb.2018.06.003 30262316 10.1016/j.jtemb.2018.06.003

[9] Moustafa S (2019) Relationship of some ultratrace elements with atherosclerosis. Zanco J Med 23:66–73. 10.15218/zjms.2019.009

[10] Militaru C, Donoiu I, Craciun A, Scorei ID, Bulearca AM, Scorei RI (2013) Oral resveratrol and calcium fructoborate supplementation in subjects with stable angina pectoris: effects on lipid profiles, inflammation markers, and quality of life. Nutrition 29(1):178–183. 10.1016/j.nut.2012.07.006 23153742 10.1016/j.nut.2012.07.006

[11] Rogoveanu OC, Mogoşanu GD, Bejenaru C, Bejenaru LE, Croitoru O, Neamţu J, Pietrzkowski Z, Reyes-Izquierdo T, Biţă A, Scorei ID, Scorei RI (2015) Effects of calcium fructoborate on levels of C-reactive protein, total cholesterol, low-density lipoprotein, triglycerides, IL-1β, IL-6, and MCP-1: a double-blind, placebo-controlled clinical study. Biol Trace Elem Res 163(1–2):124–131. 10.1007/s12011-014-0155-9 25433580 10.1007/s12011-014-0155-9 PMC4297309

[12] Weber KS, Ratjen I, Enderle J, Seidel U, Rimbach G, Lieb W (2022) Plasma boron concentrations in the general population: a cross-sectional analysis of cardio-metabolic and dietary correlates. Eur J Nutr 61(3):1363–1375. 10.1007/s00394-021-02730-w 34825958 10.1007/s00394-021-02730-w PMC8921125

[13] Barr RD, Clarke WB, Clarke RM, Venturelli J, Norman GR, Downing RG (1993) Regulation of lithium and boron levels in normal human blood: environmental and genetic considerations. J Lab Clin Med 121(4):614–619 8454944

[14] Duydu Y, Başaran N (2023) Effects of boron exposure on human reproduction and development. Curr Opin Toxicol 34:100403. 10.1016/j.cotox.2023.100403

[15] Ying X, Cheng S, Wang W, Lin Z, Chen Q, Zhang W, Kou D, Shen Y, Cheng X, Rompis FA, Peng L, Zhu LuC (2011) Effect of boron on osteogenic differentiation of human bone marrow stromal cells. Biol Trace Elem Res 144(1–3):306–315. 10.1007/s12011-011-9094-x 21625915 10.1007/s12011-011-9094-x

[16] Jin D, Lin L, Xie Y, Jia M, Qiu H, Xun K (2022) NRF2-suppressed vascular calcification by regulating the antioxidant pathway in chronic kidney disease. FASEB J 36(1):e22098. 10.1096/fj.202100625RR 34918390 10.1096/fj.202100625RR

[17] Wei R, Enaka M, Muragaki Y (2019) Activation of KEAP1/NRF2/P62 signaling alleviates high phosphate-induced calcification of vascular smooth muscle cells by suppressing reactive oxygen species production. Sci Rep 9(1):10366. 10.1038/s41598-019-46824-2 31316111 10.1038/s41598-019-46824-2 PMC6637199

[18] Arefin S, Buchanan S, Hobson S, Steinmetz J, Alsalhi S, Shiels PG, Kublickiene K, Stenvinkel P (2020) Nrf2 in early vascular ageing: calcification, senescence and therapy. Clin Chim Acta 505:108–118. 10.1016/j.cca.2020.02.026 32097628 10.1016/j.cca.2020.02.026

[19] Sogut I, Paltun SO, Tuncdemir M, Ersoz M, Hurdag C (2018) The antioxidant and antiapoptotic effect of boric acid on hepatoxicity in chronic alcohol-fed rats. Can J Physiol Pharmacol 96(4):404–411. 10.1139/cjpp-2017-0487 28898587 10.1139/cjpp-2017-0487

[20] Korkmaz M, Turkmen R, Saritas DHH, ZK, (2019) Effect of boron on the repair of osteochondral defect and oxidative stress in rats: an experimental study. Biol Trace Elem Res 187:425–433. 10.1007/s12011-018-1381-3 29869015 10.1007/s12011-018-1381-3

[21] Adhikari N, Shekar KC, Staggs R, Win Z, Steucke K, Lin YW, Wei LN, Alford P, Hall JL (2015) Guidelines for the isolation and characterization of murine vascular smooth muscle cells. A report from the International Society of Cardiovascular Translational Research. J Cardiovasc Transl Res 8(3):158–163. 10.1007/s12265-015-9616-6 25788147 10.1007/s12265-015-9616-6 PMC5105830

[22] Yıldırım E, Sezer G (2021) Clinical plasma concentration of vinpocetine does not affect osteogenic differentiation of mesenchymal stem cells. Pharmacol Rep 73(1):202–210. 10.1007/s43440-020-00153-8 32865810 10.1007/s43440-020-00153-8

[23] Wada T, McKee MD, Steitz S, Giachelli CM (1999) Calcification of vascular smooth muscle cell cultures: inhibition by osteopontin. Circ Res 84(2):166–178. 10.1161/01.res.84.2.166 9933248 10.1161/01.res.84.2.166

[24] - National Institutes of Health (2022) Boron fact sheet for health professionals. NIH Library. https://ods.od.nih.gov/factsheets/Boron-HealthProfessional/ Accessed 26 January 2024

[25] - EFSA (2018) Overview on tolerable upper intake levels as derived by the Scientific Committee on Food (SCF) and the EFSA Panel on Dietetic Products, Nutrition and Allergies (NDA). https://www.efsa.europa.eu/sites/default/files/assets/UL_Summary_tables.pdf. Accessed 26 January 2024

[26] Khaliq H, Jing W, Ke X, Ke-Li Y, Peng-Peng S, Cui L, Wei-Wei Q, Zhixin L, Hua-Zhen L, Hui S, Ju-Ming Z, Ke-Mei P (2018) Boron affects the development of the kidney through modulation of apoptosis, antioxidant capacity, and Nrf2 pathway in the African ostrich chicks. Biol Trace Elem Res 186(1):226–237. 10.1007/s12011-018-1280-7 29536335 10.1007/s12011-018-1280-7

[27] Soliman MM, Aldhahrani A, Elshazly SA, Shukry M, Abouzed TK (2022) Borate ameliorates sodium nitrite-induced oxidative stress through regulation of oxidant/antioxidant status: involvement of the Nrf2/HO-1 and NF-κB pathways. Biol Trace Elem Res 200(1):197–205. 10.1007/s12011-021-02613-5 33559025 10.1007/s12011-021-02613-5

[28] Nielsen FH, Hunt CD, Mullen LM, Hunt JR (1987) Effect of dietary boron on mineral, estrogen, and testosterone metabolism in postmenopausal women. FASEB J 1(5):394–397 3678698

[29] Hsu JJ, Tintut Y, Demer LL (2008) Vitamin D and osteogenic differentiation in the artery wall. Clin J Am Soc Nephrol 3(5):1542–1547. 10.2215/cjn.01220308 18562594 10.2215/CJN.01220308 PMC4571147

[30] Jono S, Nishizawa Y, Shioi A, Morii H (1998) 1,25-Dihydroxyvitamin D3 increases in vitro vascular calcification by modulating secretion of endogenous parathyroid hormone-related peptide. Circ 98(13):1302–1306. 10.1161/01.cir.98.13.1302 10.1161/01.cir.98.13.1302 9751679

[31] Miljkovic D, Miljkovic N, McCarty MF (2004) Up-regulatory impact of boron on vitamin D function - does it reflect inhibition of 24-hydroxylase? Med Hypotheses 63(6):1054–1056. 10.1016/j.mehy.2003.12.053 15504575 10.1016/j.mehy.2003.12.053

[32] Naghii MR, Samman S (1997) The effect of boron supplementation on its urinary excretion and selected cardiovascular risk factors in healthy male subjects. Biol Trace Elem Res 56(3):273–286. 10.1007/BF02785299 9197924 10.1007/BF02785299

[33] Sriprasert I, Hodis HN, Karim R, Stanczyk FZ, Shoupe D, Henderson VW, Mack WJ (2019) Differential effect of plasma estradiol on subclinical atherosclerosis progression in early vs late postmenopause. J Clin Endocrinol Metab 104(2):293–300. 10.1210/jc.2018-01600 30272234 10.1210/jc.2018-01600 PMC6300071

[34] Di Renzo F, Cappelletti G, Broccia ML, Giavini E, Menegola E (2007) Boric acid inhibits embryonic histone deacetylases: a suggested mechanism to explain boric acid-related teratogenicity. Toxicol Appl Pharmacol 220(2):178–185. 10.1016/j.taap.2007.01.001 17320131 10.1016/j.taap.2007.01.001

[35] Zhong H, Yu H, Chen J, Mok SWF, Tan X, Zhao B, He S, Lan L, Fu X, Chen G, Zhu D (2022) The short-chain fatty acid butyrate accelerates vascular calcification via regulation of histone deacetylases and NF-κB signaling. Vascul Pharmacol 146:107096. 10.1016/j.vph.2022.107096 35952961 10.1016/j.vph.2022.107096

[36] Shantouf R, Kovesdy CP, Kim Y, Ahmadi N, Luna A, Luna C, Rambod M, Nissenson AR, Budoff MJ, Kalantar-Zadeh K (2009) Association of serum alkaline phosphatase with coronary artery calcification in maintenance hemodialysis patients. Clin J Am Soc Nephrol 4(6):1106–1114. 10.2215/cjn.06091108 19423565 10.2215/CJN.06091108 PMC2689878

[37] Hakki SS, Bozkurt BS, Hakki EE (2010) Boron regulates mineralized tissue-associated proteins in osteoblasts (MC3T3-E1). J Trace Elem Med Biol 24(4):243–250. 10.1016/j.jtemb.2010.03.003 20685097 10.1016/j.jtemb.2010.03.003

[38] Kode A, Rajendrasozhan S, Caito S, Yang SR, Megson IL, Rahman I (2008) Resveratrol induces glutathione synthesis by activation of Nrf2 and protects against cigarette smoke-mediated oxidative stress in human lung epithelial cells. Am J Physiol Lung Cell Mol Physiol 294:L478–L488 18162601 10.1152/ajplung.00361.2007

[39] Yamada KE, Eckhert CD (2019) Boric acid activation of eIF2α and Nrf2 is Perk dependent: a mechanism that explains how boron prevents DNA damage and enhances antioxidant status. Biol Trace Elem Res 188(1):2–10. 10.1007/s12011-018-1498-4 30196486 10.1007/s12011-018-1498-4

